# Investigating Consumers’ Perceptions and Motivations Behind Edible Insects in Greece: A Grounded Theory Approach

**DOI:** 10.3390/foods14060929

**Published:** 2025-03-09

**Authors:** Anastasia Fountouli, Elena Raptou, Konstantinos Polymeros, Efthimia Tsakiridou, Theodoros Varzakas

**Affiliations:** 1 Department of Agricultural Development, Democritus University of Thrace, 68200 Orestiada, Greece; ana.fountouli@gmail.com; 2 Department of Food Science and Nutrition, University of Thessaly, 43100 Karditsa, Greece; polikos@uth.gr; 3 Department of Agricultural Economics, School of Agriculture, Aristotle University of Thessaloniki, 54124 Thessaloniki, Greece; efitsaki@agro.auth.gr; 4 Department of Food Science and Technology, University of the Peloponnese, 24100 Kalamata, Greece

**Keywords:** awareness of entomophagy, edible insect acceptance, motives behind edible insect consumption, health concerns, grounded theory

## Abstract

Edible insects constitute a healthy food source providing a sustainable alternative to traditional animal protein. The present study explored consumers’ perceptions and attitudes toward insect consumption, and defined the main motivational factors influencing public awareness and acceptance toward entomophagy. Using a qualitative research design, individual-level data were selected from a sample of 70 consumers in Greece via semi-structured personal in-depth interviews. The Grounded Theory framework was adopted to develop awareness, perception and acceptance drivers. Although the participants were knowledgeable about the usage of insects as food, the great majority demonstrated abhorrence toward entomophagy, describing feelings of disgust and repulsion. Furthermore, the respondents seemed to be reluctant towards the distribution and availability of edible insects and insect-based food options in consumer markets, whereas food safety concerns were strong as many consumers seemed to question the relevant preparation regulations. A lack of information and cultural influences were found to restrict consumers’ acceptance of entomophagy, whereas health and food safety concerns comprised an inhibiting factor in incorporating edible insects in Greek cuisine. This study emphasized the need for a holistic information plan, which will help both food businesses and consumers understand the vital role of edible insects in modern food environments.

## 1. Introduction

The growing world population is on the ministerial agenda of many nations since the need to provide enough high-quality food in a sustainable way is a challenge that will require multiple solutions to ensure food security and environmental quality [1]. Projections forecast that the population number will exceed 10 billion by 2050, exacerbating the worldwide food crisis [2]. To have the ability to satisfy the world’s feeding demands, the overall food production between now and 2050 needs to increase up to 70 per cent [3]. As a result, ‘higher levels of input and output per unit of agricultural land area are required to produce a sufficient amount of food to nourish the population’ [4], although due to the shrinking amount of arable land and diminishing freshwater supplies, this is not a feasible scenario [5,6]. Recent evidence showed that none of the nations in the world can currently achieve the essential requirements for human wellbeing, while also managing environmental preservation norms [7,8].

Intensive agriculture practices and livestock result in a variety of environmental issues, and the production of animal protein has detrimental environmental costs. These issues include the loss of natural resources, greenhouse gas emissions intensifying global warming and soil quality degradation [9,10]. Thus, it is of utmost significance to identify alternatives to traditional sources of animal protein [1] without jeopardizing the environmental integrity [4]. Multiple studies suggest edible insects to be the solution to all the aforementioned problems [11,12,13,14,15,16,17], since insect farms demand less land and energy, and compared to other agricultural outputs, the production is fast and constant [18]. Insects have unique physiological characteristics and biological structures that enable high efficiency in the conversion of protein into animal protein and feed energy into food energy with a lower environmental cost [8,19,20]. As a case in point, 3,000 times more water and 12.5 times more feed are needed to produce 1 kg of cattle in comparison to 1 kg of crickets [8]. In addition to that, insects can grow on organic waste, solving the problem of waste management and the necessity for landfills [21].

In terms of human nutrition, edible insects’ nutritional value is comparable to that of other meat sources, and from a nutritional perspective, the balance of their macro and micro components is even more beneficial in some species [22]. Edible insects constitute high energy-dense food products and are rich in antioxidants and vitamin B12 with a significant content of high-quality micronutrients, such as iron and zinc [19,23,24]. Their protein content ranges from 25% to 65% per 100 gm [25,26], and they also have a high content of mono-unsaturated fatty acids, Omega-3 and Omega-6 fatty acids [27]. However, edible insect consumption might abet health and nutrition risks associated with the anti-nutrients included, such as oxalates, cyanides, phytic acid, tannins and hydrogen, which could have serious impacts on nutrients’ absorption, digestion and usage [16,23,28]. It seems that there is still a gap in ensuring food safety, especially in countries in which edible insects are harvested from the wild [29]. Another significant barrier to edible insect consumption is the widespread perception of insects as filthy pests and carriers of zoonotic illnesses which are dangerous to human health rather than as a wholesome food source [30,31]. Following recent evidence, although insects carry viruses and can infect humans through direct blood injection, it has not been documented that these viruses can spread through consumption (i.e., ingestion) [31,32]. Recent studies [33,34] and the European Food Safety Authority evaluated the risk profile of insects used for food and feed and their toxicological characteristics indicating that both humans and vertebrate animals are at low risk from viruses [35].

The consumption of edible insects among human societies has a lengthy and opulent past. Evidence of people eating insects can be found in prehistoric archaeological sites, dating back thousands of years [36]. Even though on the continents of Africa, Asia, America, Australia and the country of New Zealand people have consumed insects for centuries [37] in ancient Greece, eating insects was quite uncommon. To a certain degree, however, some historical records reference the utilization of specific insects as food for humans and feed for animals. For instance, Aristotle mentioned that caterpillars were consumed in rural areas, and they were used as fishing bait [38,39]. Furthermore, in the History of Animals, Aristotle mentioned the consumption of cicadas [40]. In more recent years, bee brood appeared to be a delicacy in the northern part of Andros Island, where locals used to include raw to well-cooked bee brood in their diet, a practice that took place up until the 1960s and has been abandoned ever since [40].

Although eating insects is increasingly becoming more popular on a global scale with over two billion people consuming them regularly [19], attitudes, perceptions and motivations for entomophagy vary greatly between countries. A great body of the literature has paid attention to a combination of cultural, psychosocial and behavioural factors, which influence individuals’ dietary patterns and shape the drivers of insect-based food acceptance [30,41,42,43,44,45,46]. Recent evidence showed that in nations with an established culinary heritage, such as Greece, France and Italy, customers were hesitant to try novel foods that they were not traditionally incorporated into their diet in comparison to the South and Latin America [47] and other Western counties, such as Denmark, Belgium and the Netherlands [48,49]. In Denmark, edible insect-based foods were widely advertised on the media resulting in a greater consumer familiarity with entomophagy and its benefits and a higher acceptance of insect-based products in the supermarkets [50]. Similarly, consumers in Belgium were positive in relation to finding insect-based products in the supermarkets, particularly energy bars and sandwich spreads, where the insect ingredients were invisible in food [51]. Comparably, in Finland, customers were more likely to buy products where the insect visibility was low, and the insect was either ground or incorporated into the dish [52,53]. On the contrary, in France and Ireland, food neophobia and feelings of aversion were linked to entomophagy, with customers being unwilling to consume insects that were visible and not incorporated into foods [54]. In Italy, insects were perceived as a threat [42], and consumers who expressed neophobic reactions were less likely to try edible insects. According to Moruzzo et al. [55], edible insect-based foods were also perceived as disgusting, and consumer acceptance of entomophagy depended on an array of factors, such as the species of the insect, whether the insect would be visible or incorporated into the food, its life-stage and the type of the dish [56]. Similarly, evidence from Poland showed that feelings of neophobia and disgust were predominant, and psychological barriers averted consumers from switching to alternative meat substitutes, such as insects [57].

Edible insects and insect-based foods are not yet available in Greek food markets. A recent study noted that Greek consumers conveyed feelings of repulsion and disgust and seemed to reject the usage of insects as food options [46]. However, negative attitudes could be moderated if insects were incorporated into the food products and became unnoticeable to the customers. With respect to the young adult consumer segment, Kamenidou et al. [45] demonstrated that although Gen Zs seemed rather familiar with the aspects of entomophagy, young adults were unwilling to replace meat protein with insect protein and engage in insect consumption. Communication strategies were deemed necessary to increase Gen Zs’ awareness and provide information about the entomophagy benefits for both human health and environmental ecosystems [45,58]. The present study sought to investigate consumers’ perceptions and attitudes towards entomophagy in Greece using qualitative data from a widespread national sample. It also explored individuals’ awareness of edible insect and insect-based food products and the motivational factors that could help increase people’s willingness to try edible insects. Furthermore, the directions of insect-based foods’ acceptability were investigated in order to curb the barriers that restrict the adoption of edible insects in Greek consumers’ dietary patterns.

## 2. Materials and Methods

### 2.1. Theoretical Framework of the Study

The present study employed a qualitative research design to delve into the factors influencing individuals’ attitudes and perceptions towards entomophagy and clarify the mechanism of consumer choice. Qualitative research approaches are employed to address the complexities of human behaviours and explore the social interactions in order to provide a clear understanding of the “real world issues” [59]. Qualitative approaches are also used when a concept has neither been developed before nor is deemed sufficient and when objective phenomena provide an inadequate explanation [60,61]. Furthermore, qualitative methods, such as in-depth interviews, can be more effective than quantitative approaches in exploring complex phenomena. In comparison with quantitative research designs, qualitative methods seek to disclose the description and interpretation of various phenomena, without measuring or testing associations, or trying to build statistical models [62]. According to Agius [62], “qualitative research normally takes an inductive approach, moving from observation to hypothesis rather than hypothesis-testing or deduction”. Given that entomophagy is not practised by the Greek population and edible insect-based food options are not available through the food channels, the qualitative approach was considered as the most appropriate to explore the factors determining individuals’ awareness, perceptions and acceptance of edible insect consumption. Following Stull et al. [63] who explored the social acceptability of edible insects in rural Zambia, the Grounded Theory framework was adopted to accomplish the study goals. Qualitative methods have been widely applied in current food research [64,65,66,67,68], whereas the Grounded Theory approach allows researchers to investigate relationships and processes, which can explain and shape consumers’ behavioural intentions and decisions [69].

Huston and Rowan [70] defined the Grounded Theory as the “generation of a theoretical model through the experience of observing a study population and developing a comparative analysis of their speech and behavior”. According to Creswell [71], the Grounded Theory comprises an appropriate tool for building an explanatory model, surpassing the mere description of a scenario. Since the Grounded Theory methodology is inductive, it is employed to investigate relationships attempting to explain why an event becomes apparent or why people may behave in a certain way [61,72]. Under the Grounded Theory assumptions, data are subjected to a continuous examination until the theory construction related to the research goals is developed [73]. Using an inductive logic, the research is not structured under specific research hypotheses to prove (or disprove), but rather follows a more straightforward process including data collection and their concurrent analysis before generating research hypotheses [74]. An analytical presentation of the Grounded Theory approach is provided by Bryant [75].

### 2.2. Sample Selection

Theoretical sampling techniques were adopted to select the participants from all over Greece, representing various sociodemographic profiles [76,77]. The sample sizes in qualitative research can be rather small for achieving saturation, especially in homogenous populations [78,79,80,81,82]. However, a larger number of participants may help researchers better depict behavioural patterns by exploring a greater variety of perceptions and experiences, and also augment the possibilities of accomplishing data saturation [83,84,85,86].

The sample size was set by using the inductive thematic saturation model as a standard for when to cease further data analysis. Following qualitative research fieldwork, the analysis of responses is key to what is known as inductive thematic saturation: when the emergence of new themes and new codes has plateaued. [87,88]. Compared to quantitative research, in which the intent is to secure a large random sample, which is representative of the total population, in qualitative research “generalisability is based on the assumption that it is valuable to begin to understand similar situations or people, rather than being representative of the target population” [62].

Since there is a great diversity of eating patterns and food habits across Greece, we employed a sample of 70 participants, considering the necessity for a large-scale nationwide survey to ensure participation from all over the country (covering both the Greek mainland and the islands), to obtain an in-depth understanding of the topic of interest. Greece has a very rich gastronomic culture and tradition with significant differences across the country. For instance, “hohlioi boubouristoi” (snails in a skillet with olive oil, vinegar and rosemary) comprises a traditional Cretan recipe, although it is quite unpopular in the rest of the country.

### 2.3. Data Collection and Analysis

Data were collected through personal in-depth interviews with selected individuals who expressed their interest in the study after seeing a Facebook advertisement post inviting participation in the study. A semi-structured questionnaire was outlined to understand individuals’ perceptions, attitudes, feelings and thoughts [89]. The interview questions were open-ended and conveyed themes, such as participants’ awareness of entomophagy and familiarity with edible insects, as well as perceptions of eating edible insects and processed insects as a whole, or incorporating isolated edible insect components into food products. We also assessed individuals’ attitudes towards edible insect-based foods, the elements influencing consumption choice and purchase decisions, and viewpoints on the prospect of making edible insect-based food products available to the market through the food supply chain (i.e., grocery shops, supermarkets and restaurants). The questions included were pre-defined, but participants could redirect the interview and add new information through their interaction and discussion with the interviewee [63]. The main questions are presented below:

- “Have you ever heard of the term entomophagy?”
- “Are you aware of the benefits of eating edible insects?”
- “Have you ever eaten edible insects before?”
- “If yes, in what form (raw, cooked, ground, or incorporated in food)?”
- “What are your feelings towards entomophagy?”
- “What are the images and thoughts that come to your mind when thinking of edible insects?”
- “Are you willing to try edible insects?”
- “Are you willing to try food that has edible insects incorporated into it?”
- “What are your reasons to try edible insects?”
- “How do you find the idea of having edible insects sold in grocery stores and having them incorporated into the menu of restaurants in the future in Greece?”

The interviews were tape recorded and performed on Skype, and their duration was approximately 30–40 min. The qualitative data selection process was initialized on November 2024, and lasted one month. All the participants were made aware of the study’s specifics and were ensured personal data confidentiality. Participation was optional and voluntary, and the participants were free to leave at any time with no repercussions. Furthermore, there were no time restrictions, and the respondents were free to share their experiences, thoughts and perceptions. Ethical approval was provided by the Ethics Committee of the Democritus University of Thrace (DUTH/EC/23903/192).

**Figure 1:**
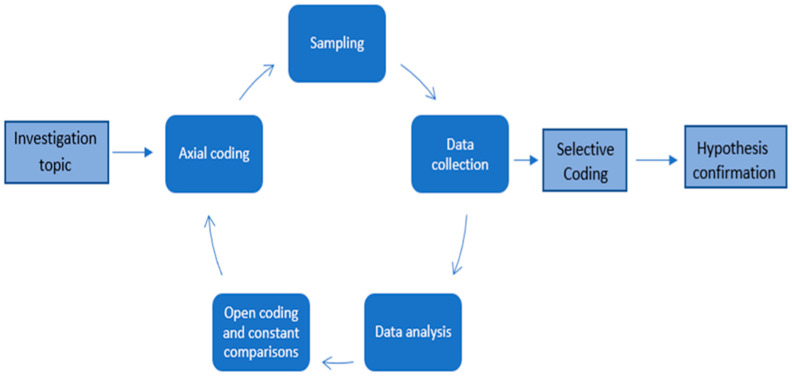
Data analysis process.

The data analysis, as described in Figure 1, was implemented according to the Grounded Theory framework [73,90,91,92,93].

Data were analyzed using the three-stage methods of open coding, axial coding and selective coding [94]. During the open coding stage, a sub-category was created for codes that had a similar notion; these were the coding units [95]. During the axial coding step, the relationships between categories and between a category and its subcategories, were defined. The axial coding procedure eventually established each category’s characteristics [96]. During the selective coding stage, a central category was chosen, and the other categories that were connected to it and the others were then combined to establish and improve the theoretical background [97,98]. Comparisons were employed throughout to identify the characteristics of each notion, and all the analyses were conducted in accordance with the raw data. Data saturation was achieved, and the theoretical construction was completed when the new added information failed to create new categories [98].

## 3. Results

### 3.1. Participants’ Profile

**Figure 2:**
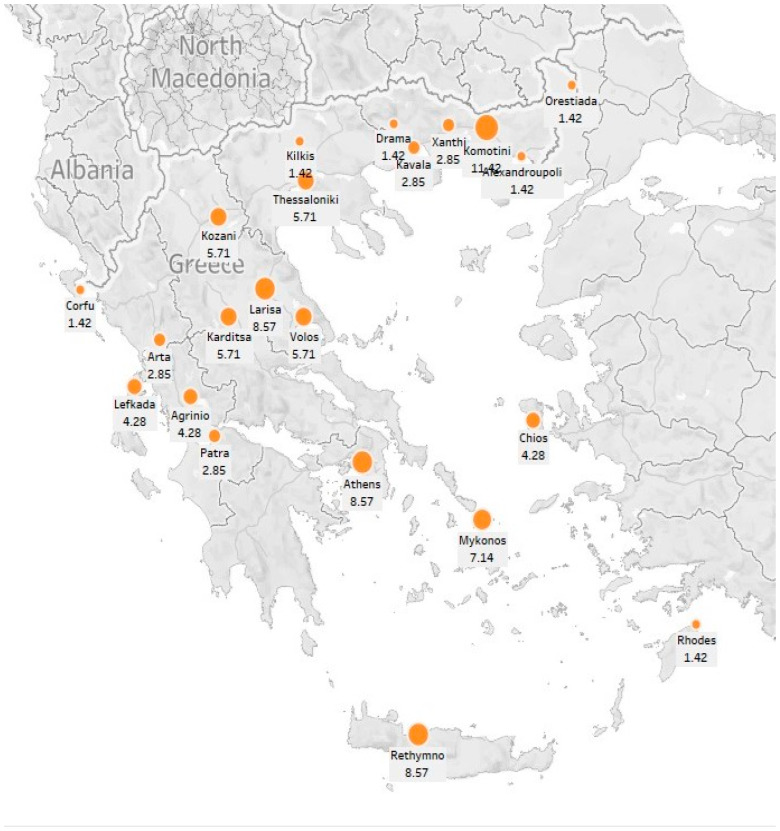
Participants’ area of residence % (*N* = 70).

The sample participants presented a diverse range of demographic characteristics, including age, educational attainment and area of residence. In particular, 70 individuals participated in the interviews, of whom 30 were male (43% of the sample) and 40 were female (57% of the sample). The respondents’ age varied between 18 and 72 years with a mean age of 39 years. With regard to their educational level, 47% were university graduates, 27% had completed the basic secondary education and 26% were graduates of a Technical Educational Institute. Furthermore, 77% of the participants resided in the Greek mainland and the remaining 23% in the islands (Figure 2 presents, in detail, the participants’ area of residence). The participants’ monthly average income was EUR 900. Fish was a valuable source of animal protein for the 96% of the sample, followed by beef and chicken (94% of the sample), seafood (93% of the sample), turkey (89% of the sample) and pork (86% of the sample).

### 3.2. Grounded Theory Results

Participants’ responses were organized based on thematic categories. In total, three main categories (MCs) were developed with ten generic categories (GCs) and twenty-nine codes that showed the frequency of statements based on the participants’ awareness, perception and acceptance of edible insects. The three MCs corresponded to:

- Consumers’ awareness of entomophagy (MC1);
- Consumers’ perceptions of entomophagy (MC2);
- Consumers’ acceptance of edible insects (MC3).

The data abstraction process and the thematic categories developed from the analysis of the participants’ responses are presented in Appendix A.

#### 3.2.1. Participants’ Awareness of Entomophagy

**Figure 3:**
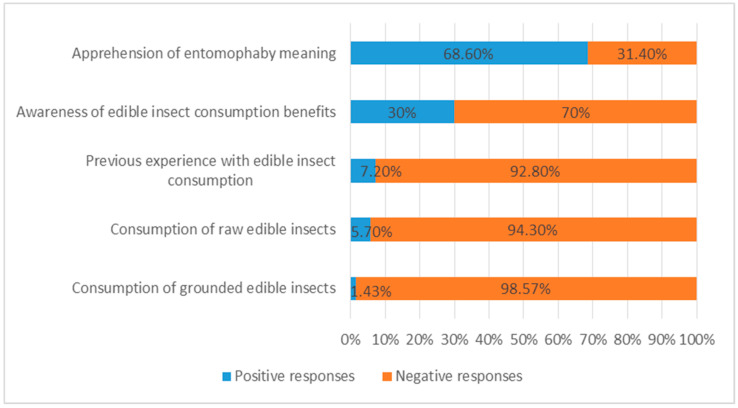
Participants’ awareness of entomophagy (*N* = 70).

The participants demonstrated their awareness of edible insect consumption by explaining how they perceived entomophagy, entomophagy practices, and their potential benefits in human diet and health. Additional questions on previous eating experience with entomophagy and the type of edible insect consumed (raw, cooked, ground or incorporated into food) sought to provide a thorough understanding of consumers’ awareness of the implementation of edible insects in Greek cuisine. The main findings are summarized in Figure 3. The great majority of the participants responded positively when the question “Have you ever heard of the term entomophagy?” was asked. A great proportion of the respondents claimed that they were either acquainted with its meaning or perceived the term as self-explanatory due to its Greek root. Furthermore, almost equal proportions of male and female participants were familiar with the concept of entomophagy. More specifically, over half of both genders reported having been informed through social media, magazine articles, peers, TV advertisements and documentaries, although the information acquired seemed to be limited and somewhat vague.

Some of the participants’ responses are presented below:

“Yes, I have heard this term before on the news and social media. More specifically, there was an article on Facebook that was detailing which insects have been approved for human consumption” (Participant 2, female, 59 years old).

“I have heard this term before; it is about the consumption of edible insects. Lately, I have seen it mentioned in various articles online, some (people) were for it and some others were against it” (Participant 5, female, 65 years old).

“No, I have never heard of this term before. However, I have watched some documentaries on TV where people in China ate insects, so I assume this might be it?” (Participant 1, male, 36 years old).

Obliviousness was expressed by the majority of the sample (code: Unaware) in regard to their awareness of the edible insect consumption benefits. Several of the participants mentioned that they had never heard of any benefits before, expressing their doubts as to whether there is any positive contribution of entomophagy to health and nutrition. Furthermore, suspicion and hesitation were displayed among a significant proportion of the respondents in terms of the potential benefits of edible insect-based food consumption. Their feedback is provided below:

“Even if the government imposes entomophagy on us, I think there are no benefits at all, and fake research results will be published to convince us that there are” (Participant 25, male, 24 years old).

“No, I don’t think there could be any benefits at all” (Participant 55, male, 41 years old).

“I have never been informed, and I really don’t care” (Participant 26, male, 34 years old).

A minor proportion of the respondents expressed feelings of impartiality and detachment, showing a lack of motivation to try edible insects, or even learn about their nutritional content and their health aspects.

“I am not aware of any potential benefits but even if there were, entomophagy is such a strange and unfamiliar concept to me that those benefits would never convince me to adopt entomophagy and include edible insects in my everyday diet” (Participant 34, male, 41 years old).

“I would never eat insects; hence, I have never searched for any potential benefits that they may have. For instance, I do not like green peas so what is the point of researching for their benefits if I am not going to eat them?” (Participant 36, male, 36 years old).

On the contrary, 30% of the sample responded positively and raised opinions of apprehension regarding the advantages of edible insect consumption (code: Aware). Some of them considered edible insects a good source of protein, noting that they could help reduce world hunger, either consumed raw or processed:

“Good source of protein. I’ve seen documentaries that showed gorillas eating ants for this very reason” (Participant 21, male, 24 years old).

“As far as I am concerned edible insects are rich in fatty acids and protein. I read somewhere in an online article that their nutritional value is compared to the one of orange juice and olive oil” (Participant 56, male, 38 years old).

“I am not very informed but I am under the impression that the consumption of edible insects will alleviate world hunger” (Participant 29, female, 33 years old).

When the participants were asked if they had ever eaten edible insects before, only a small proportion of them (5 out of the 70 participants) mentioned a past experience, declaring that they had tried raw insects in salads while on a trip abroad or ground in cereal bars and flour.

“Yes, I have eaten insects in cereal bars. You cannot tell the difference in flavour, they were delicious” (Participant 3, male, 64 years old).

#### 3.2.2. Participants’ Perceptions of Entomophagy

**Figure 4:**
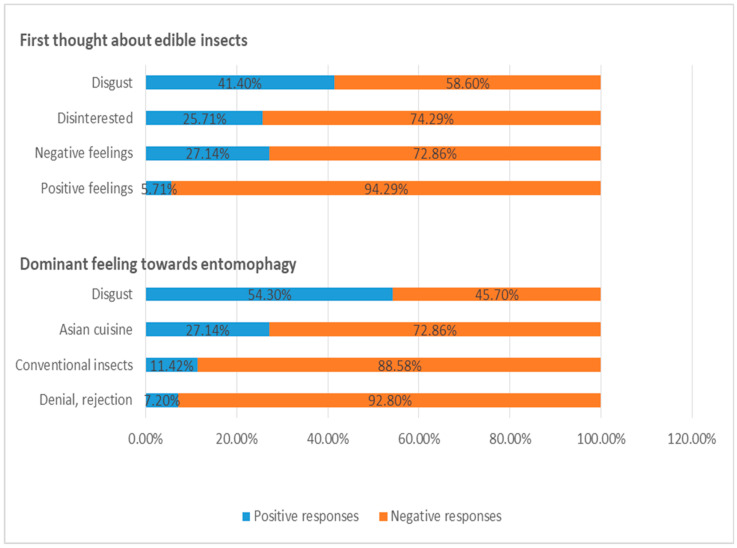
Participants’ perceptions of entomophagy (*N* = 70).

The participants’ perceptions of entomophagy were expressed through a description of their dominant feelings towards edible insect consumption and through sharing the first thoughts on the prospect of “eating bugs”. The main findings are summarized in Figure 4.

Of the 70 participants, 29 (41.4% of the sample) described senses of disgust, aversion and nausea as their dominant feelings towards entomophagy (code: Disgust). Nineteen participants (27.14% of the sample) showed negative feelings of disappointment, embarrassment and frustration towards consuming edible insects (code: Negative), whereas approximately 26% of the sample mentioned either a lack of interest in insect-based foods or mistrust as to how insect-based components are implemented in processed foods (code: Disinterested). The respondents’ relevant expressions are reported below:

“I do not have certain feelings about the concept of entomophagy. Unlike other countries where entomophagy is immersed in their culture, in Greece we do not consume edible insects. Thus, I haven’t put much thought into it” (Participant 7, female, 23 years old).

“I have negative feelings but I do not object to anyone who consciously chooses to eat edible insects. However, I am not in support of the misinformation and misapprehension surrounding the subject, i.e., I am opposed to the addition of substances derived from edible insects into food. People are not aware that these substances are implemented into our diet. They may be included on the food labels but they are given fancy names that are unknown to the public. Ultimately, we do not know what we are eating” (Participant 35, female, 52 years old).

However, only a small number of the participants expressed positive feelings and satisfaction towards entomophagy, underlining the necessity for an effective food control system to assure the safety and quality of insect-based food products (code: Positive). For instance, the following statements were provided:

“I am willing to try edible insects if they were approved and certified as a food option” (Participant 9, female, 28 years old).

“I have positive images of the food in Chinese markets where they sell bug kebabs (insect skewers)” (Participant 12, female, 29 years old).

When the participants were asked about the thoughts/images that they evoked on entomophagy, over half of them expressed disgust and aversion, mixing up edible insect food components with dirty, unhealthy or inappropriate food constituents (code: Disgust). However, a few respondents explained that their apparent curiosity concerning such types of newly introduced foods might overcome any other obstacles, motivating them to experiment with entomophagy and processed edible insect-based foods.

“I am disgusted but curious at the same time about their flavour. I would try edible insects because I like challenges” (Participant 1, male, 36 years old).

“I am angry because they try to force entomophagy as something natural when it is not. Humans should not be eating insects” (Participant 36, male, 36 years old).

In addition, edible insect consumption seemed to entail cultural dimensions. Thus, a notable proportion of the respondents considered entomophagy a dietary style influenced by the Asian culture and cuisine, and also believed that it could not be adapted to the dietary habits and preferences established in Greek society (code: “Asian cuisine”).

“I think of something salty, crunchy and tasty like crisps. Something to whet your appetite. It would potentially go well with cocktails” (Participant 12, female, 29 years old).

However, a few participants highlighted the perceived delicacy of cooked and processed edible insects, claiming that they could comprise delicatessen food products for special dinners and social gatherings.

“Asian food markets, a Chinese habit, a foreign concept in comparison to our diets” (Participant 16, female, 31 years old).

Several of the participants also noted that they unwittingly restrain themselves from exploring new/novel food items, and therefore edible insects could never comprise for them an alternative food source. For this specific consumer segment “insects are just insects” (code: Insects). Furthermore, a small group of the respondents mentioned that they were opposed to entomophagy and were reluctant to share their thoughts about edible insects (code: Denial).

“I refrain from eating edible insects and will continue to do so in the future. I have no images and thoughts to share” (Participant 15, male, 30 years old).

#### 3.2.3. Participants’ Acceptance of Entomophagy

**Figure 5:**
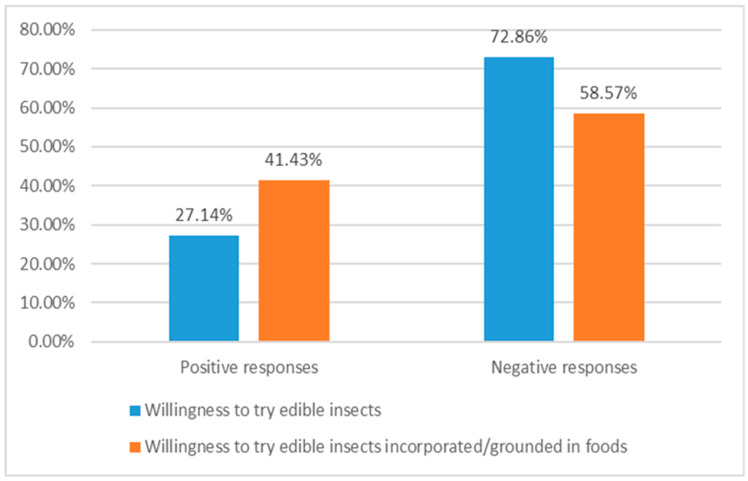
Participants’ willingness to try edible insects (*N* = 70).

The participants’ acceptance of entomophagy was explored through individuals’ willingness to try/taste edible insects (either raw or incorporated/ground in food items), their motivations towards entomophagy engagement and their perceptions on the prospect of edible insects being distributed through supermarkets and grocery stores, and also being incorporated into the restaurant menus. The main findings showed that a significant proportion of the respondents (approximately 27%) seemed proponents to entomophagy, explaining that they would rather try edible insects in the near future (Figure 5). Furthermore, the share of consumers revealing attitudes in favour of edible insect consumption was even higher in the case of food items with incorporated/ground insects or insect constituents (approximately 41.4%). However, the great majority of the sample respondents seemed convinced that they would never eat edible insects unless during a travel experience or due to specific medical conditions. When the participants were asked if they would be willing to include edible insects in their dietary routine in a future-time perspective, all of the sample participants expressed negative views, mostly annoyance and frustration.

Some of the participants’ statements are listed below:

“No, not in Greece but perhaps if I was on a trip in Asia” (Participant 13, female, 26 years old).

“No, I consider insects disgusting and I do not even think of eating them—unless I was under a specific medical treatment” (Participant 36, male, 36 years old).

“Yes, but it depends on the type of insect. For instance, I cannot fathom the idea of eating insects that have an exoskeleton” Participant 7, female, 23 years old).

“Yes, if the insects were incorporated in food and I was not aware of it” (Participant 17, female, 42 years old).

“I am willing to eat edible insects if the insect is hidden and not seen. Preferably, if the insect was ground it would make the transition to an edible insect diet easier” (Participant 7, female, 23 years old).

**Figure 6:**
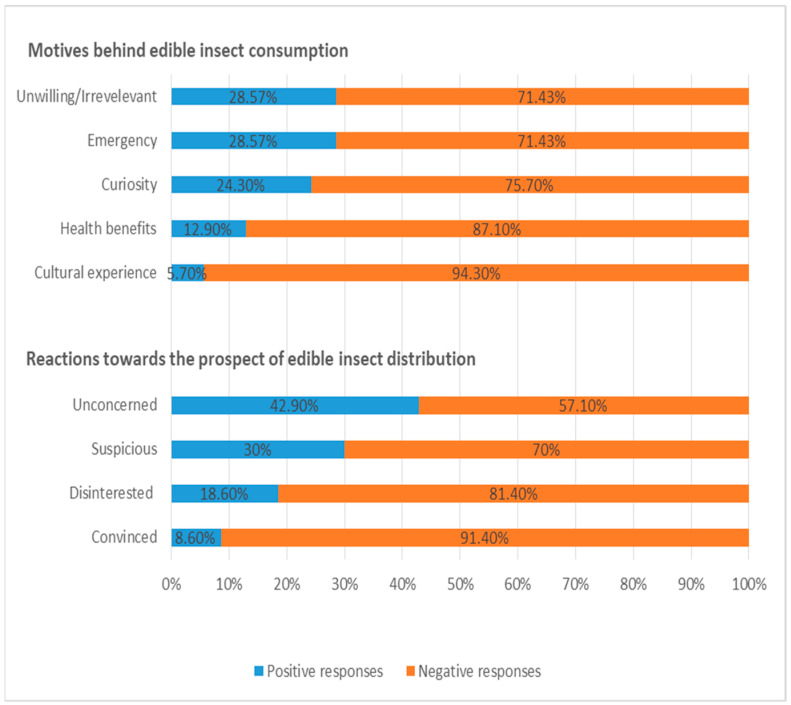
Motives behind edible insect consumption and participants’ reactions towards edible insect distribution through the food system (Ν = 70).

With respect to consumers’ motivations for adopting entomophagy, a significant proportion (approximately 29% of the sample) disclosed their unwillingness, explaining that they felt awkward even in the thought of trying edible insects, and also questioned the hygiene and safety of insect-based foods (code: Unwilling/Irrelevant) (Figure 6). However, a significant share of the respondents attested that they could adapt to entomophagy under a state of emergency (i.e., war or hunger), although adopting a plant-based dietary pattern (e.g., vegan) instead would constitute a more reachable option (code: Emergency). More specifically, they professed:

“Edible insects are an unsuitable food option for humans” (Participant 30, male, 33 years old),

“This is an irrelevant question; I have already said that I am disgusted. I will feel sorry for myself if we reach that low point in life where edible insects will be part of our diet in Greece” (Participant 52, female, 41 years old).

“If I was very hungry or we were under war” (Participant 20, male, 44 years old).

“If we were in a state of emergency but if I am being honest, I would much rather convert to veganism than eat insects” (Participant 23, female, 56 years old).

Several of the participants also agreed that curiosity about the taste, smell and texture of the edible insects would comprise a strong motive for their consumption, noting that it would be best if they were served cooked (code: Curiosity):

“I am curious and that is the only reason why I would ever try edible insects. Having said that though, I would never substitute meat for insects. I like the taste of meat” (Participant 1, male, 36 years old).

“I am curious about their flavour. In France, people eat frog legs and snails, which is not much different than eating edible insects, right?” (Participant 45, female, 38 years old).

Furthermore, medical reasons and anticipated health benefits constituted additional motivations for edible insect consumption (code: Benefits), whereas expectations for new cultural experiences also stimulated the participants’ interest in entomophagy (code: Cultural experience). For instance, the participants said:

“If something is beneficial to human health then I see no reason why we should not try it. However, Greece is a country where the majority of its citizens are superstitious and do not believe in science so I think it will be difficult to convince them to eat bugs, even in case there were real health benefits” (Participant 56, male, 38 years old).

“As part of a cultural experience. Also, if they were served in a gourmet, fine dining restaurant” (Participant 7, female, 23 years old).

When the participants were asked how they would react at the prospect of edible insects being distributed through the food system in Greece, and also incorporated into restaurant menus, there was a noticeable variation in their responses, which were categorized under four different codes. In particular, almost one-third of the respondents seemed unconcerned in regard to that perspective, noting that it could not cause them any discomfort or anxiety, especially if edible insects were marketed in smart packaging and placed on separate shelves in food stores (code: Unconcerned).

“As long as they were sold in packages, then I would not have a problem with it” (Participant 44, female, 38 years old).

On the contrary, a significant proportion of the sample participants expressed distress about edible insect distribution through the food chain in the near future, declaring that they would never support businesses selling “bugs” to customers (code: Distressed).

“I am not happy about it. I would rather move to another country than shop from grocery stores where bugs are sold” (Participant 55, male, 41 years old).

“I would be devastated if this were to happen and I would boycott businesses that sell edible insects” (Participant 70, female, 45 years old).

Several of the participants seemed to perceive edible insect sales as an unrealistic prospect, considering infeasible their implementation in the Greek diet and cuisine. Furthermore, the respondents were suspicious about the safety and the hygiene of edible insect-based food options, underlining the necessity to build sophisticated food systems with control standards that meet international requirements (code: Suspicious):

“I do not care if this will happen in the future, but I think it is insane. Grocery stores pay a lot towards making sure their stores are disinfected, so why sell insects?” (Participant 8, female, 22 years old).

Lastly, only a few participants (8.6%) seemed to support the development of edible insect distribution systems in the food environment, emphasizing the impacts of the modern agricultural production and livestock systems on natural resources, and the need to introduce more environmentally friendly food options to account for the rising environmental concerns (code: Convinced).

“Intensive agriculture and farming have detrimental effects on the environment, so consuming edible insects in the future comes with no surprise to me. I would be willing to try foods with edible insects but only if they were not very expensive. For instance, I would not buy an expensive cake that was made out of insect flour” (Participant 1, male, 36 years old).

“I think this possibility is very much going to happen shortly. It takes a lot of energy to produce mincemeat, so I am hoping that one day we will eat insect burgers that are much more environmentally friendly, in comparison” (Participant 29, female, 22 years old).

#### 3.2.4. The Influence of Gender on Perceptions and Edible Insect Acceptance

Gender was the only sociodemographic indicator that was found to have a statistically significant influence on consumers’ behaviour towards entomophagy. In particular, female respondents were more likely to present positive attitudes and perceptions towards edible insect consumption compared to their female counterparts (27.5% vs 6.7%, chi-square test = 4.920, *p*-value = 0.027) (Table 1). Furthermore, women were also more likely to accept entomophagy, whereas men seemed more reluctant towards edible insect consumption (52.5% vs 26.7%, chi-square test = 4.715, *p*-value = 0.026) (Table 2).

## 4. Discussion

Despite the continued efforts by experts to promote edible insects as an environmentally friendly source of protein, our study found that entomophagy is not widely accepted among the consumers in Greece with only a small segment of the participants willing to try edible insects—a finding that is consistent with other studies focusing on the acceptance of entomophagy in both Greece and other European countries [45,55,99,100,101,102]. Edible insect consumption is not practised, as insect-based goods are not available in the market; therefore, consumers lack previous experience and display a limited familiarity with entomophagy. Furthermore, individuals’ readiness to eat insects may be affected by neophobic tendencies and their attention to the environmental impact of food [99,103,104,105]. Lack of knowledge and consumers’ feelings of disgust can lead to an overall negative attitude that requires a carefully designed strategy to change consumers’ well-rooted beliefs. Many people in Western societies are disgusted by the prospect of eating insects and by the insect itself [48], despite the fact that insects constitute a valuable food source in many cultural regions around the world [106]. The presence of insects might even be considered as an indicator for low hygiene since insects have been considered as an indicator for food contamination and health risks [107]. Thus, if an individual is disgusted by poor food hygiene, then this could become a significant predictor for the willingness to eat products made with processed insects [108].

### 4.1. Participants’ Awareness of Entomophagy

The ability of consumers to comprehend the concept of entomophagy and understand the term, even if they had not eaten insects before, was reported by Halonen et al. [53]. The participants of the current study displayed an accurate assumption of the concept of entomophagy, even though a limited number of them practised it, due to the fact that the word entomophagy is derived from the Greek language. Understanding the term came as a natural occurrence to the majority of the participants, but an overall pessimistic attitude heralded our findings subsequent to the unappealing idea of consuming edible insects. The reasons are multi-faceted. In Greece, many idioms and proverbs refer to insects, generating negative feelings and, thus, influencing consumers’ food choices. Once people are exposed to repetitive linguistic conditions, their beliefs change, and they express conscious feelings of disgust [109,110]. The high percentage of consumers diverging from consuming edible insects could also be due to insect-based dishes clashing with the traditional Greek flavours and are not welcomed, or even accepted, by their peers. Celebrity endorsement, peers’ opinions and message framing are all factors determining the food choices that people make and cannot be overlooked [111,112]. The same attitude applies among other European countries where insect acceptance has not scaled [53,57] due to food preferences, cultural distaste for entomophagy and insufficient legislation [41,99,113,114,115], despite them being available in the market.

The lack of consumers’ previous experience with edible insects is attributed to the fact that insect-based goods are not yet available in the food industry in Greece. As the participants were not exposed to insect-based goods and dishes, they illustrated obliviousness to the nutritional value of edible insects reaching the point of ignorance. Kamenidou et al. [45] in Greece, reported similar findings with none of their study’s participants engaging in entomophagy, which is in line with Orsi et al. [30] in Germany and Lorini et al. [42] in Italy, demonstrating the need for proper public education to enhance the awareness of edible insects’ nutritional characteristics.

The participants were more likely to opt for edible insects and edible insect-based goods if the insect was cooked and/or ground, as they viewed it less challenging when the novel ingredient is incorporated into a dish. Orkusz et al. [57] in Poland and Halonen et al. [53] (2022) in Finland reported similar findings as they claimed that there is a higher chance for consumers’ disposition in relation to entomophagy to increase, when the insect is in a processed form (ground or cooked) and undetectable. However, it is not just the insect visibility, or lack thereof, that controls consumers’ ability to accept entomophagy. Consumers with a poorer educational background are also less prone to buy edible insects, although in the context of the present study, the highly educated participants still declined the opportunity to practise entomophagy, contradicting previous studies claiming that consumers with an extensive educational background are inclined to eat novel foods [99,116,117,118,119].

Consumers in Western countries prefer conventional animal to insect-based protein [120,121], a notion that matches the beliefs of consumers in Greece who, regardless of the benefits of insect-based foods, hold that insects are not a food choice [45]. It appears that the participants are yet to accept entomophagy which highlights the need to educate the public about their diet’s carbon footprint and the nutritional benefits of consuming edible insects. Furthermore, it may be critical to examine marketing strategies from a consumer psychology angle [112] that are employed to promote alternative protein.

### 4.2. Participants’ Perceptions of Entomophagy

The possibility of entomophagy in Greece is frowned upon, as it is in many other Western nations [30,122] with negative preconceptions [103,123], leading to consumer rejection and resulting in negative emotions, denial and apathy on the subject. The consumers in our study did not consider edible insects as a primary food source or as a feed ingredient, potentially due to the lack of knowledge regarding the benefits and a total incomprehension of the safety of insect-based goods. Feelings of aversion and disgust were communicated leading to a rejection of edible insects for fear of food poisoning, sickness and unpleasantness, with the participants claiming that pests are disease vectors. On the whole, the idea of practising entomophagy was either disturbing or generated feelings of refusal. These findings on the participants’ perception of entomophagy are consistent with those of other studies [49,124,125,126,127,128,129] where the participants described almost identical feelings towards entomophagy, profoundly demonstrating the need for consumers’ education on the matter and the design of a clear legal framework on edible insects’ consumption in Greece that would reassure and appease consumers.

### 4.3. Participants’ Acceptance of Entomophagy

Food neophobia is a significant obstacle to the integration of novel foods including insect-based food options in the human diet [45,46,48]. Consequently, when the participants of our study faced questions that included an unfamiliar food option, fear dominated their acceptance. Considering that entomophagy is not practised in Greece, and the barriers to the acceptance of edible insects by consumers are many, our findings are interesting due to the partial acceptance of entomophagy on the condition that the insect would be ground and not on display, and/or that it would occur as part of a cultural experience during a trip abroad. Ostensibly, it is clear that the prospects of accepting entomophagy in Greece rely on the manner in which a dish or product is presented, insect clarity in food and the cultural circumstances in which the dish is prepared and served. In this context, an establishment of a series of culinary products made out of ground edible insects coupled with a positive messaging marketing promotion would have the prospect of consumers’ appeal in Greece following the example of the Dutch [121]. It is also important to target certain consumer groups that are more likely to accept, taste and endorse these food options to their peers, as when consumers enjoy novel food options, this can lead to approval and over exploration [130]. In all likelihood, these groups are consumers who have had a past experience with entomophagy and are more inclined to consume edible insects again. Lensvelt and Steenbekkers [131] concluded the same for consumers in Australia and the Netherlands. Informed consumers changed their perception of the practice. A strategy’s likelihood of success in one nation may not apply to another; however, psychological motivation needs to be considered to include insects in the Greek diet.

The participants had no intention to alter their pattern of animal protein consumption and had a strong belief that edible insects would never be sold in the Greek market with a small percentage favouring the idea. On account of this, their reasons for trying edible insects would be curiosity and being in a state of emergency. The potential health benefits were another driver of acceptance as it has also been reported in the past, that the health benefits of different kinds of food influence behaviours more than views [118]. Hedonistic eating values surpass utilitarian eating values by a wide margin as the participants believe that insects have no utilitarian aspect, a finding that was also reported by Norwegian consumers [128]. Hedonic values control consumer decision making [132]; therefore, for edible insect foods to be commercially successful in Greece, the food industry needs to increase its hedonic value, and also inform the public about the health benefits that they bring to the table. The production of confectionary products, snacks and appetizers that contain edible insects, such as cereal bars made out of ground edible insects, and insect chips strongly flavoured with spices is likely to triumph in the Greek market. In conclusion, it is important to educate consumers in Greece about entomophagy in the broadest sense while understanding consumers’ food preferences to increase the acceptance of entomophagy. Attitudes must change for consumers to make informed decisions; therefore, there is a deep necessity for public education.

### 4.4. Study Limitations

The study has some limitations. The study participants had to discuss fictitious foods and dishes while eliciting their valuations, since insect-based products are not available in Greece. It is still possible that our participants experienced hypothetical biases even though we followed all the recommended steps outlined in the literature to account for or lessen them. Future studies combining both qualitative and quantitative research designs could provide an integrated picture of consumers’ attitudes and perceptions towards edible insects and the motivations behind entomophagy.

## 5. Conclusions

The current study provides a new understanding of consumers’ perceptions and motivations behind entomophagy in Greece based on a theoretical framework that analyzes the components impacting individuals’ insights and strives to define the means of consumers’ options. Given the abundance of studies in other countries suggesting that edible insects are the liberators of famine and a considerable source of protein [133,134,135,136,137], this study stretches the argument by encompassing social and psychological factors, such as the participants’ past experiences with edible insects, and their thoughts and feelings and how these are intertwined with their beliefs. Focusing on Greece, a country where the research on this topic remains limited since entomophagy is not practised and consumers are unfamiliar with edible insects, this study also offers knowledge on consumers’ perceptions by providing their reactions to edible insects’ availability in the Greek market.

In Greece, a small share of the food market product abundance is dedicated to delicacies from the “Ethnic Cuisine” category. These widely accepted commodities (noodles, tortillas, sauces and dips) are advertised as “exotic” and are endorsed by celebrities, influential figures and chefs of TV cooking shows demonstrating them to the public eyes as ordinary and everyday products in a supportive way. Had it not been for the seal of approval and enormous influence of the mass media, consumers would have not accepted them. That being the case, it is apparent that a collective movement should take place with the mass media, social media, and hospitality and food industries involved altogether to alter the negative beliefs that consumers in Greece have towards edible insects and allow them to welcome this unconventional protein source into their diet. It appears that even though edible insects are not the traditional animal protein source that Greek consumers would choose, with the appropriate messaging and advertisement, edible insects have the potential to be incorporated into the consumers’ diets and to reach many Greek households. Public awareness campaigns that use online advertisements, TV cooking shows and targeted social media posts should be materialized to reach all audiences and groups and contribute to the cause.

The employment of academics, health specialists, doctors and dieticians to broadcast their knowledge on the benefits of consuming edible insects, and the health advantages and environmental implications of eating alternative proteins is another step in potentially forming the first segment of consumers that would adopt entomophagy. This could be achieved by educational initiatives, such as teaching relevant courses at schools, distributing posters with nutritional facts across all hospitals and healthcare centres in the country, and by having recipes, that include edible insects as their main ingredient, available at the food markets. To arouse curiosity, free food samples that include edible insects as one of their ingredients could also be handed out to consumers at supermarkets and food courts. These trials could be a way to convert people into loyal customers of the product.

The key step, however, to establishing entomophagy in Greece is to make it known to the public that edible insects are safe for human consumption. Reassuring consumers that there are no detrimental effects caused by edible insects’ ingestion is crucial for the acceptance of entomophagy in Greece. This could be achieved through transparent food safety regulations that highlight the nutritional benefits of insect-based diets. Health professional endorsements could also further enhance consumer trust and encourage a shift in perception toward edible insects as a viable and beneficial food source. Within the relevance of this work, future research should be conducted to popularize the study’s results, develop strategies and involve stakeholders to allow for the best approach to entomophagy acceptance in Greece while meeting consumers’ demands and expectations.

## Figures and Tables

**Table 1:** Respondents’ gender and perceptions towards entomophagy (*N* = 70).

Perceptions Towards Entomophagy		Male	Female	Chi-Square Test	*p*-Value
Negative perceptions	% within perceptions	49.1%	50.9%	4.920	0.027
	% within gender	93.3%	72.5%		
Positive perceptions	% within perceptions	15.4%	84.6%		
	% within gender	6.7%	27.5%		

**Table 2:** Respondents’ gender and acceptance of entomophagy (*N* = 70).

Acceptance of Entomophagy		Male	Female	Chi-Square Test	*p*-Value
Rejection	% within perceptions	53.7%	46.3%	4.715	0.026
	% within gender	73.3%	47.5%		
Acceptance	% within perceptions	27.6%	72.4%		
	% within gender	26.7%	52.5%		

## Data Availability

The original contributions presented in this study are included in the article. Further inquiries can be directed to the corresponding authors.

## Appendix A

**Table A1:** Thematic categories developed from data abstraction (*N* = 70).

Main Categories (MC)	Generic Categories (GC)	Codes
Awareness	Familiarity with the meaning of entomophagy	Yes (n = 48)
		No (n = 22)
	Awareness of edible insect health benefits	Aware (n = 21)
		Unaware (n = 49)
	Past experience	Yes (n = 5)
		No (n = 65)
	Form of edible insect consumption	Raw (n = 4)
		Grounded (n = 1)
Perceptions	First thought on edible insects	Disgust (n = 38)
		Asian cuisine (n = 19)
		Insects (n = 8)
		Denial (n = 5)
	Dominant feeling towards entomophagy	Disgust (n = 29)
		Disinterested (n = 18)
		Negative (n = 19)
		Positive (n = 4)
Acceptance	Willingness to try edible insects	Yes (n = 19)
		No (n = 51)
	Willingness to try food products with incorporated edible insects	Yes (n = 29)
		No (n = 41)
	Motives behind edible insect consumption	Unwilling/Irrelevant (n = 20)
		Emergency (n = 20)
		Curiosity (n = 17)
		Health benefits (n = 9)
		Cultural experience (n = 4)
	Reactions towards the prospect of edible insect distribution	Unconcerned (n = 30)
		Suspicious (n = 21)
		Distressed (n = 13)
		Convinced (n = 6)
